# Inactivated Whole Virus Influenza A (H5N1) Vaccine^1^

**DOI:** 10.3201/eid1305.061248

**Published:** 2007-05

**Authors:** Zoltan Vajo, Lajos Kosa, Ildiko Visontay, Mate Jankovics, Istvan Jankovics

**Affiliations:** * National Center for Allergy and Immunology, Budapest, Hungary;; † National Center for Epidemiology, Budapest, Hungary; and; ‡ Semmelweis University Medical School, Budapest, Hungary

**Keywords:** influenza, H5N1, vaccine, human, clinical trial, letter

**To the Editor:** Avian influenza viruses of the H5N1 subtype represent a potential source of the next pandemic (*1*,*2*). Our goal was to determine the safety and immunogenicity of a newly developed vaccine in humans.

The vaccine was produced by the same method as the interpandemic influenza vaccine “FluvalAB” used in Hungary for the past 11 years (*3*,*4*). The method has been validated by meeting the requirements of the European Agency for the Evaluation of Medicinal Products (EMEA) related to interpandemic influenza vaccines each year since 1995, and by having been administered in humans in a total of >15 million cases (*5*).

The virus strain (NIBRG-14), a reverse genetics–derived 2:6 reassortant between A/Viet Nam/1194/2004 (H5N1) and PR8, was obtained from the National Institute for Biologic Standards and Control, London. It is one of the reference viruses indicated as suitable for use in a mock-up vaccine by the Committee for Medicinal Products for Human Use (*6*).

Hens’ egg–grown, formaldehyde-inactivated, whole virus vaccine, developed and produced by the Omninvest Ltd. (Budapest, Hungary), was used. The vaccine contained 6 μg hemagglutinin per dose (as determined by single radial immundiffusion test) in 0.5-mL ampules. Purity was assessed by endotoxin content (determined by chromogenic endotoxin assay, using a modified limulus amoebocyte lysate and a synthetic color-producing substrate), which was considered acceptable in concentrations <0.1 IU/mL. The amount of ovalbumin was determined by ELISA, which was considered satisfactory in concentrations <10 ng/mL. Aluminum phosphate was used as adjuvant, in the amount of 0.31 mg Al per ampule; 0.1 mg/mL merthiolate was added as preservative.

A total of 146 healthy volunteers >18 years of age (mean ± SD 42.07 ± 12.62 years). were enrolled in the study. Sixty-five male and 81 female volunteers participated. The sample size was chosen to exceed the requirement of 50 patients per group set by the European guidelines for yearly influenza vaccine trials (*5*). The sponsor was the National Public Health and Medical Officer Service, Budapest, Hungary.

The injection administered 0.5 mL of vaccine intramuscularly. The injection was not repeated. Serum antibody titers were measured by hemagglutination inhibition (HI) by using chicken erythrocytes, following standard procedures (*7*). Because the protective titer for influenza virus A (H5N1) infections is unknown, immunogenicity was assessed according to the European Medicines Agency criteria related to interpandemic influenza vaccines (Table) (*5*).

**Table:** Immunogenicity findings of whole-virus influenza vaccine trial, Hungary*†

	CHMP‡ requirement‡	Total study population	Male	Female
				
Day 21				
GMT	NA	27.9	31.0	25.6
Post- to pre-vaccination GMT ratio (increase)	>2.5	5.6‡	6.2‡	5.1‡
% of participants seropositive (titer >1:40)	>70	63.7	70.8‡	58.0
% of participants with seroconversion (4-fold titer increase or titer >1:40)	>40	63.7*	70.8‡	58*
Day 90				
GMT	NA	29.4	31.9	27.4
Post- to pre-vaccination GMT ratio (increase)	>2.5	5.9‡	6.4‡	5.5‡
% of participants seropositive (titer >1:40)	>70	6.3	73.9‡	61.8
% of participants with seroconversion (4-fold titer increase or titer >1:40)	>40	67.3‡	73.9*	61.8‡

*CHMP, Committee for Medicinal Products for Human Use, European Medicines Agency; GMT, geometric mean titer; NA, not applicable. †Hemagglutination-inhibition (HI) titers below the limit of detection were given an arbitrary value of 1:5. GMTs of antibody and their confidence intervals were computed by transforming the results to a logarithmic scale, assuming asymptotic normality conditions were satisfied on the scale and converting back to the original scale. HI endpoints were the GMT at each timepoint and the variables required for interpandemic influenza vaccines: postvaccination seropositivity rate (% of participants with titers ≥40), the post- to pre-vaccination GMT ratio, and the proportion of persons seroconverting or displaying a 4-fold titer increase postvaccination.: ‡Met CHMP standards.

None of the study participants displayed measurable levels of HI antibodies before vaccination. According to EMEA requirements, both male and female groups met 2 independent criteria for immunogenicity 21 and 90 days after vaccination (Table).

In 15.7% of the participants, adverse reactions in the form of local pain at the injection site occurred within the first 48 hours; these reactions disappeared within 1 day. No other local reactions, such as injection site induration, erythema, swelling, warmth, or ecchymosis, were noted. No systemic reaction (fever, malaise, headache, shivering) was detected. No serious adverse events were observed. These results are in line with the 11-year experience using the interpandemic vaccine produced by Omninvest Ltd. by the same method, where a similar safety profile has been seen after >15 million vaccinations in humans.

This is the first study that reports that an inactivated whole virus vaccine with an aluminum phosphate adjuvant system against influenza A (H5N1) was safe and immunogenic in humans after only 1 injection. This study reports the lowest effective dose used to cause immune response. Other trials used much higher maximum doses and required 2 injections 21 or 28 days apart (*8*–*10*). Using the lowest possible amount of the antigen and fewer injections is essential for increasing the production capacity of vaccine manufacturers in a pandemic (*2*).

Using 1, instead of 2, injections will shorten the time needed to develop immune response by 3–4 weeks. Unlike previous studies on influenza A (H5N1) vaccines that reported only data from 21, 28, or 56 days after the final vaccination (*8*–*10*), we report data up to 90 days. The lower dose and fewer injections required to trigger an immune response can be at least partially explained by using a whole virus vaccine and an aluminum phosphate adjuvant system. The use of a different adjuvant system than ours may have influenced the results of other trials (*9*,*10*). Other investigators used a modified HI method with horse erythrocytes, which are known to be more sensitive for influenza A (H5N1) subtype than the conventionally used turkey or chicken erythrocytes (*8*,*9*). Thus, if horse erythrocytes had been used in our study, the vaccine would likely have been even more immunogenic.

This study found fewer, less frequent, and milder side effects than did other trials of influenza A (H5N1) vaccines published so far (*8*–*10*). This could possibly be explained by the smaller dose used. Also, the endotoxin content of 0.1 IU/mL in our vaccine was much smaller then the allowed amount of 100 IU/mL by standards (*5*).

We report an inactivated whole virus vaccine that is safe and immunogenic in healthy adults and that requires a low dose and only 1 injection to trigger an immune response. We are conducting trials in elderly persons and children.

## References

[1] Ungchusak K, Auewarakul P, Dowell SF, Kitphati R, Auwanit W, Puthavathana P, et al. Probable person-to-person transmission of avian influenza A (H5N1). N Engl J Med. 2005;352:333–40. 10.1056/NEJMoa044021 15668219

[2] Dennis C. Flu-vaccine makers toil to boost supply. Nature. 2006;440:1099. 10.1038/4401099a 16641963

[3] Takatsy G. Purified precipitated virus obtained by a new simple method. Acta Med Acad Sci Hung. 1952;3:185–91. 13007362

[4] License number: OGYI-T-8998/01. Budapest: National Institute of Pharmacology; 1995.

[5] European Committee for Proprietary Medicinal Products. Note for guidance on harmonization of requirements for influenza vaccines, March 1997 (CPMP/BWP/214/96). Brussels: European Agency for the Evaluation of Medicinal Products; March 12, 1997.

[6] European Committee for Proprietary Medicinal Products. Guideline on dossier structure and content for pandemic influenza vaccine marketing authorisation application (CPMP/VEG/4717/03). Brussels: European Agency for the Evaluation of Medicinal Products; April 5, 2004.

[7] Klimov A, Cox N. Serologic diagnosis of influenza virus infections by hemagglutination inhibition. Influenza laboratory course. Atlanta: Centers for Disease Control and Prevention; 2003.

[8] Treanor JJ, Campbell JD, Zangwill KM, Rowe T, Wolff M. Safety and immungenicity of an inactivated subvirion influenza A (H5N1) vaccine. N Engl J Med. 2006;354:1343–51. 10.1056/NEJMoa055778 16571878

[9] Bresson JL, Perronne C, Launay O, Gerdil C, Saville M, Wood J, Safety and immunogenicity of an inactivated split-virion influenza A/Vietnam/1194/2004 (H5N1) vaccine: phase I randomised trial. Lancet. 2006;367:1657–64. 10.1016/S0140-6736(06)68656-X 16714186

[10] Lin J, Zhang J, Dong X, Fang H, Chen J, Su N, Safety and immunogenicity of an inactivated adjuvanted whole-virion influenza A (H5N1) vaccine: a phase I randomised controlled trial. Lancet. 2006;368:991–7. 10.1016/S0140-6736(06)69294-5 16980114

